# Extraordinary MHC class II B diversity in a non-passerine, wild bird: the Eurasian Coot *Fulica atra* (Aves: Rallidae)

**DOI:** 10.1002/ece3.974

**Published:** 2014-02-13

**Authors:** Miguel Alcaide, Joaquin Muñoz, Josué Martínez-de la Puente, Ramón Soriguer, Jordi Figuerola

**Affiliations:** 1 Estación Biológica de Doñana – CSICAvda. Américo Vespucio s/n, 41092, Sevilla, Spain; 2 The University of Oklahoma Biological Station15389 Station Road, Kingston, Oklahoma, 73439

**Keywords:** Adaptive genetic variation, concerted evolution, gene copy variation, Immunogenetics, pathogen-mediated selection

## Abstract

The major histocompatibility complex (MHC) hosts the most polymorphic genes ever described in vertebrates. The MHC triggers the adaptive branch of the immune response, and its extraordinary variability is considered an evolutionary consequence of pathogen pressure. The last few years have witnessed the characterization of the MHC multigene family in a large diversity of bird species, unraveling important differences in its polymorphism, complexity, and evolution. Here, we characterize the first MHC class II B sequences isolated from a Rallidae species, the Eurasian Coot *Fulica atra*. A next-generation sequencing approach revealed up to 265 alleles that translated into 251 different amino acid sequences (*β* chain, exon 2) in 902 individuals. Bayesian inference identified up to 19 codons within the presumptive peptide-binding region showing pervasive evidence of positive, diversifying selection. Our analyses also detected a significant excess of high-frequency segregating sites (average Tajima's D = 2.36, *P* < 0.05), indicative of balancing selection. We found one to six different alleles per individual, consistent with the occurrence of at least three MHC class II B gene duplicates. However, the genotypes comprised of three alleles were by far the most abundant in the population investigated (49.4%), followed by those with two (29.6%) and four (17.5%) alleles. We suggest that these proportions are in agreement with the segregation of MHC haplotypes differing in gene copy number. The most widespread segregating haplotypes, according to our findings, would contain one single gene or two genes. The MHC class II of the Eurasian Coot is a valuable system to investigate the evolutionary implications of gene copy variation and extensive variability, the greatest ever found, to the best of our knowledge, in a wild population of a non-passerine bird.

## Introduction

The major histocompatibility complex (MHC) hosts the most polymorphic genes ever described in vertebrates (e.g., de Bakker and Raychaudhuri 2012; Bollmer et al. 2012). MHC genes trigger the adaptive branch of the immune system by binding and presenting short microbial peptides (antigens) to lymphocyte T cells (see reviews by Sommer 2005; Piertney and Oliver 2006). The astonishing variability exhibited by MHC genes has been commonly attributed to the extraordinary diversity of pathogens in the environment. For instance, the more MHC alleles an individual harbors, the larger the diversity of pathogens that individual is expected to cope with (“heterozygosity advantage” hypothesis; e.g., Oliver et al. 2009; Penn et al. 2002). However, several studies have suggested that in some cases individuals with an intermediate number of MHC alleles (“optimality” hypothesis) might have higher fitness (e.g., Wegner et al. 2003; Kloch et al. 2010; Stiebens et al. 2013), probably because of lower chances to generate autoimmune disease due to self-reacting lymphocytes (see Milinski 2006; Woelfing et al. 2009). Species thriving in spite of reduced or a lack of MHC variation have questioned whether MHC diversity is explicitly critical to counteract microbial infections (e.g., Radwan et al. 2010; Gangoso et al. 2012), and this finding suggests that certain alleles could play a key role regulating infections (e.g., Kloch et al. 2010; Westerdahl et al. 2011). The loss of rare alleles by random drift is also believed less accentuated at the MHC, as natural selection may confer them a selective advantage against emerging pathogens (“frequency-dependent selection” hypothesis; see reviews by Sommer 2005; Spurgin and Richardson 2010). Spatiotemporal variations in pathogen-mediated pressures (Hill 1991) and disassortative mating preferences (e.g., Juola and Dearborn 2012; Strandh et al. 2012; but see Havlicek and Roberts 2009; Westerdahl 2004) are also believed to increase MHC variability.

Traditionally, MHC genes have been broadly classified in two main types according to the origin of the antigens presented (extracellular for the MHC class II and intracellular for the MHC class I, reviewed by Sommer 2005). However, current evidence supports that some degree of cross-presentation occurs (e.g., Cresswell et al. 2005; Iwasaki and Medzhitov 2010). The release of extensive genomic resources from model organisms, the accumulation of sequence data from non-model species, and the advent of next-generation sequencing NGS (reviewed by Babik 2010) have greatly facilitated the access to MHC loci in a wide diversity of species. Birds are not an exception to this general trend, and the last few years have witnessed a substantial release of avian MHC sequences (e.g., Ekblom et al. 2004; Alcaide et al. 2007, 2013; Westerdahl 2007; Burri et al. 2008; Hughes et al. 2008; Zagalska-Neubauer et al. 2010; Li et al. 2011; Strandh et al. 2011; Sepil et al. 2012). These studies have nevertheless unraveled important differences among different avian groups, not only regarding the complexity of the multigene families but also with respect to their extent of genetic variability and mode of evolution (see for instance Burri et al. 2008; Gangoso et al. 2012; Bollmer et al. 2012;.) Generally, the MHC of songbirds (Aves: Passeriformes) is more complex in terms of gene duplicates and diversity than nonpasserine birds (e.g., Westerdahl 2007).

Here, we report what we believe the largest extent of MHC class II diversity ever reported in a non-passerine wild bird so far, the Eurasian Coot *Fulica atra* as revealed by a next-generation sequencing approach. Our study is also the very first to isolate MHC sequences in a Rallidae species (coots, rails, gallinules, and allies) and therefore, fills this gap in the evolutionary history of MHC genes in birds. Given their life history characteristics, coots are exposed to a large diversity pathogens, including water, food, and air-transmitted virus, bacteria, helminths, and other parasites (Schistosomiasis, *Pasteurella multocida*,*Mycobacterium avium*,*Campylobacter sp*., *Salmonella sp.,* or Adenovirus; Hubálek 2004; Cromie et al. 2012). These birds therefore emerge as excellent model systems to investigate the evolutionary implications of MHC variation on pathogen-mediated selection. In addition, recent studies have highlighted the relevance of Eurasian Coots as reservoirs of the West Nile virus in the Mediterranean basin. Populations of coots have been intensively monitored, and high prevalence of antibodies has been reported in Spain, Czech Republic, India or Iran (Figuerola et al. 2007; Hubálek et al. 2008; Fereidouni et al. 2011; Mishra et al. 2012), without detectable cases of disease despite the high exposure to the virus (López et al. 2011). Hence, this study may lay the foundations for the examination of the role of MHC variation on the efficiency of the immune response in these birds.

## Materials and Methods

### Birds capture and blood sampling

In this study, we included samples from a total of 952 Eurasian Coots. Birds were trapped without damage using walk-in-traps from 1999 to 2012 in the Cañada de los Pájaros (Seville, Spain; 6°14′W, 36°57′N), near the Doñana National Park (SW Spain). Birds were marked with numbered aluminum rings, and blood samples were taken from the tarsal vein using syringes. The volume of blood extracted never exceeded 1% of avian body mass. A drop of each blood sample was maintained in absolute ethanol and frozen (−20°C) until molecular analyses. Kinship relationships were unknown. Given the large population size of Eurasian coots in the area, usually more than 10,000 individuals as suggested by annual aerial census (unpublished data), we expect our sample to be mostly comprised of unrelated birds.

### Isolation of MHC class II B genes in the Eurasian Coot

Genomic DNA was isolated from blood samples using a standard protocol (Gemmel and Akayama 1996) or the DNeasy Blood and Tissue® kit (QIAGEN, Hilden, Germany). MHC class II B genes, which encode for one of the subunits of the dimeric MHC class II molecule, were isolated through a traditional approach relying on the polymerase chain reaction (PCR). First, a genomic fragment spanning exon 1 to exon 2 was amplified using primers MHC05 (Miller and Lambert 2004) and 325 (Ekblom et al. 2003) in six individuals. The PCR protocol consisted of a cycle of 3 min at 95°C, followed by 34 cycles of 60 s at 95°C, 40 s at 60°C, and 40 s at 72°C, with a final step of 10 min at 72°C. PCR amplifications were carried out in 20 *μ*L total volume containing 2 *μ*L of 10X PCR buffer (Bioline, London, UK), 2.5 mM of MgCl_2_ (Bioline), 0.60 *μ*M of each primer, 250 *μ*M of each dNTPs, and 0.6 U Taq DNA polymerase (Bioline) and about 10 ng of genomic DNA. Partial MHC fragments were directly sequenced according to the BigDye 3.1 terminator technology and resolved into an ABI3730xl automated sequencer (Applied Biosystems, Foster City, CA). We designed a new primer Fuat-Ex2Fw 5′-CTGACCRGCCTCCCTGCA-3′ sitting on a conserved 3′ distal region of intron 1, as suggested by the alignment of the sequences obtained from the six birds (a consensus sequence of that region has been deposited as supplementary information (Data S1). Second, a genomic fragment spanning exon 2 to exon 3 was amplified using the primers Fuat-Ex2Fw 5′-CTGACCRGCCTCCCTGCA-3′ and RapEx3CR primer (Alcaide et al. 2007) in 15 individuals. The PCR and sequencing protocol were the same than described above. A reverse primer Fuat-Ex2Rv 5′-TTGTGCCAYACACCCACC-3′ sitting on a conserved region of the beginning of intron 2, as suggested by the alignment of the sequences of the 15 birds (see consensus sequence in Data S1), was then designed. The two coot-specific primers successfully amplified a fragment of the expected size (around 300 bp after agarose gel electrophoresis) in 15 individuals using the same PCR protocol described above. Our primers amplify the entire coding sequence of exon 2 (270 bp), which is known to host the vast majority of sites under selection within the MHC class II peptide-binding region (PBR), plus the last seven nucleotides of intron 1.

### 454 Library preparation and analysis

We designed fusion primers consisting of a 454/GS FLX Titanium adapter sequence + a barcode sequence ranging from 4 to 8 nucleotides (all barcodes differed in at least 3 nucleotides) + the Eurasian Coot MHC class II B specific primers. As up to 32 FW and 32 RV tagged fusion primers were available (i.e., 32 × 32 = 1024 possible combinations), each of the 952 coots could be tracked by a unique barcode combination. Here, the PCR protocol was the same than that for the original Fuat-Ex2Fw and Fuat-Ex2Rv primers but with a couple of modifications aimed at minimizing the impact of chimera formation (Lenz and Becker 2008). First, the number of cycles was decreased to 24 cycles, and the polymerase extension steps were increased to 4 min per cycle. The amplification products were electrophoresed in a 2% agarose gel, and quantification was performed using the Quantity One 1-D Analysis software (Bio-Rad, Hercules, CA) before creating a pool roughly containing equimolar concentrations for each individual. The pool was purified using Agencourt AMPure XP beads (Beckman Coulter, Brea, CA) and quantified fluorometrically with the Quant-it Picogreen dsDNA Assay Kit (Invitrogen, Carlsbad,CA). This pool was amplified in an emulsion PCR using the GS Junior Titanium emPCR kit Lib A, (Roche Applied Science, Penzberg, Germany), and pyrosequencing was carried out on a 454 GS Junior System using the Sequencing Method Manual GS Junior Titanium Series following manufacturer's instructions (454 Life Sciences, Branford, CT).

Handling and manipulation of the obtained 454 runs were accomplished using the commercial software Geneious, version 6.1.6 (Drummond et al. 2011). First, sequences were demultiplexed according to their barcode combination. Second, the reads obtained from each individual were assembled using the low sensitivity de novo assembly settings of Geneious, with a couple of modifications. Here, maximum mismatches among reads were set at 2% (to account for PCR and sequencing errors), and the maximum gap size allowed was set at six nucleotides. This approach is capable to sort out the sequences of alleles differing just in one nucleotide position when co-occurring in the same individual if both alleles are represented by a substantial number of sequences. Basically, this method outputs a series of contigs that summarizes the presumptive set of different sequences within a given individual. We then aligned the 75% threshold consensus sequences from each contig and grouped them by similarity using the MUSCLE multiple aligner plugin (Edgar 2004) implemented in Geneious. We checked that consensus sequences did not contain ambiguities that could be related to the inclusion of closely related alleles within the same contig. The number of reads comprising each contig was annotated in the consensus sequence using the Find Low/High coverage option implemented in Geneious (e.g., if one contig was comprised of 50 similar or identical sequences, the consensus for that assembly displays a value of 50). Next, we searched for blocks of identical consensus sequences isolated from different individuals or barcode combinations and with a minimum of 12 reads. These blocks of sequences were named as a particular allele (e.g., Fuat-DAB* + allele number, following the nomenclature proposed by Klein et al. 1990). We provisionally accepted those alleles found in one single bird but with a reasonable number of reads (minimum = 12 reads) as long as the percentage of reads for that allele was not blatantly below putative co-occurring alleles, and the allele did not resemble a chimeric sequence. Chimeric sequences add indeed serious difficulties to the genotyping of complex MHC multigene families involving the simultaneous amplification of large numbers of alleles (e.g., Zagalska-Neubauer et al. 2010; Sommer et al. 2013). The Eurasian coot MHC class II B was not problematic regarding chimeric sequences because the vast majority of individuals (>70%, see below) displayed three or less alleles, there usually was a largely homogeneous representation for each allele within a given individual and anything else was clearly under-represented and easy to identify as an artifact. Finally, the list of putative alleles was set as a local database to run custom BLAST in Geneious. The consensus sequences from the contigs obtained for each individual were compared with this database. Geneious then provides a table from which we could retrieve those alleles from the database showing a 100% match hit as well as the number of reads representing each allele in each individual.

### Genetic analyses

**Figure 1:**
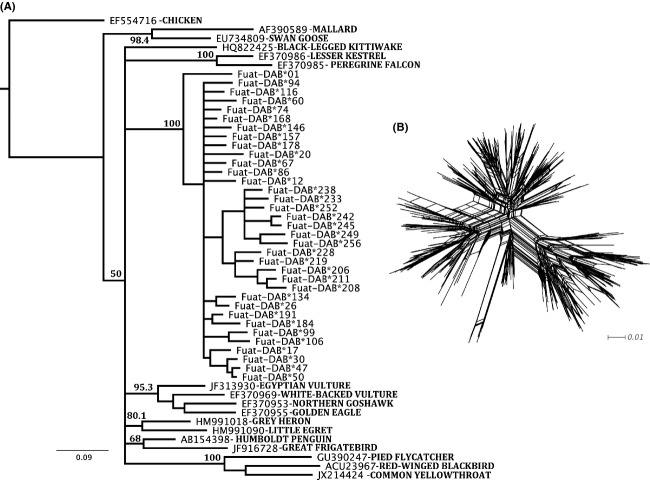
(A) Neighbor-joining tree of a random subset of Eurasian Coot MHC class II B exon 2 alleles plus a few homologous avian sequences download from the public domain (GenBank Acc, Nos. are provided in the figure). Bootstrap values based on 1000 replicates are given. (B) Phylogenetic network of 265 exon 2 alleles isolated from the Eurasian Coot. Tips represent different alleles.

Polymorphism statistics at the set of isolated MHC class II B sequences were generated using the software DNAsp, version 5.0 (Librado and Rozas 2009). We also used DNAsp to conduct a Tajima's D-test (Tajima 1989) on the obtained repertoire of exon 2 alleles. Functional and evolutionarily relevant MHC sequences are commonly characterized by an excess of nonsynonymous (*d*_n_) over synonymous (*d*_s_) substitution rates at codons belonging to the PBR, which is a signature of positive, diversifying selection (then *ω* = *d*_n_/*d*_s_ > 1) in response to selection imposed by pathogens. Site-by-site tests of selection were conducted through a Fast Unconstrained Bayesian AppRoximation (FUBAR) that uses a Markov Chain Monte Carlo (MCMC) routine as implemented in the Datamonkey web server (http://www.datamonkey.org/). This method has been suggested to be more statistically robust and fast than previous methods to infer selective patterns along coding sequences (Murrell et al. 2013). Because recombination is critically believed to overestimate the number of positively selected sites (e.g., Anisimova et al. 2003), we first tested for significant evidence of recombination along our alignment of exon 2 sequences. We used the single breakpoint recombination (SBP) method, (Kosakovsky Pond et al. 2006) also implemented in the Datamonkey web server. The original alignment was subsequently subdivided into different partitions when both the AIC and the BIC criterion reported statistically significant evidence of recombination breakpoints. The nucleotide substitution model best fitting to our sequence alignment was selected using the corresponding tool implemented in the Datamonkey web server. We also carried out a test of positive selection using the software MEGA 5.0 (Tamura et al. 2011). For this analysis, we conducted a priori classification of PBR versus non-PBR codons according to the crystallographic structure of the human MHC class II molecule (Brown et al. 1988). Synonymous and nonsynonymous substitutions rates were calculated through the modified Nei–Gojorobi method (Nei and Gojobori 1986) with Jukes–Cantor correction, and variance estimation was based on 1000 bootstrap replicates. Finally, the phylogenetic relationships among the Eurasian Coot MHC class II B alleles were visualized through a phylogenetic network built in the software Splitstree 4.0 (Huson and Bryant 2006). Their relationship with regard to other avian MHC class II sequences downloaded from the public domain (see Fig. 1 for species identity and GenBank Acc. Nos) was visualized through a neighbor-joining tree built from HKY genetic distances (Hasegawa et al. 1985) in Geneious.

## Results

We gathered more than 250,000 reads through pyrosequencing analysis. From the 952 Eurasian coots analyzed, 50 were discarded due to an insufficient number of 454 reads (<25). The average number of reads across the remaining 902 individuals was 283.9 ± 284.1 SD (range: 29–2741). Number of alleles was positively correlated with number of reads, but only very weakly (*r* = 0.075, *P* = 0.024). The analysis of these 902 birds revealed 266 unique nucleotide sequences (GenBank Acc. Nos. KF924770-KF925035) corresponding to 265 unique exon 2 sequences that translated into 251 putatively functional amino acid sequences. The allele Fuat-DAB*199, isolated from a unique bird but with a high number of reads (*N* = 57), displayed a stop codon and was excluded from further analyses. This allele could represent a PCR artifact occurring during the very first replication steps or a nonfunctional allele present at a very low frequency in the population. Up to 203 alleles were retrieved from at least two different birds or independent typing experiments of the same bird. Sixty-three alleles were only isolated from one single bird in the population. Fifteen birds were amplified twice with different barcode combinations and analyzed in independent 454 runs, for which we obtained a 100% genotype reproducibility adding robustness to our typing method.

**Figure 2:**
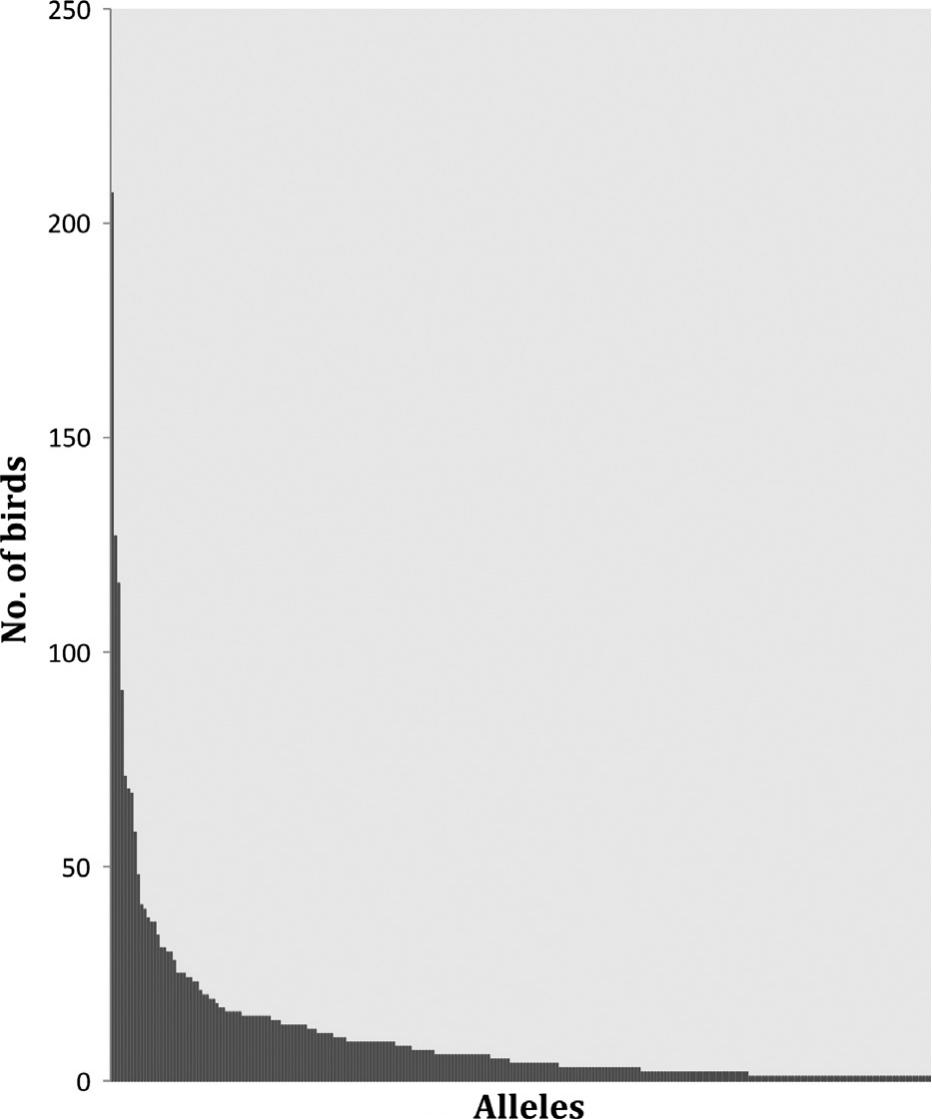
Number of individuals (Y axis), where a particular exon 2 allele amino acid sequence, (X axis), was found.

A rough estimation of the allele frequency distribution (based on the number of birds from which a particular amino acid sequence was retrieved) is given in Fig. 2 (see also Supplementary File 2). We kept the nomenclature of the most common nucleotide allele when dealing with synonymous alleles (i.e., those alleles with identical amino acid sequence but different nucleotide sequence). The three most commonly found amino acid sequences were those of Fuat-DAB*109 (*N* = 207 birds, 22.9%), Fuat-DAB*08 (*N* = 127 birds, 14.1%), and Fuat-DAB*177 (*N* = 116 birds, 12.8%). Synonymous alleles were found only in pairs (*N* = 9 pairs) except for the commonest amino acid sequence of Fuat-DAB*109, from which up to six different nucleotide sequences were isolated. We found one to six different alleles per individual, which is consistent with the occurrence of at least three MHC class II B duplicates. Interestingly, the genotypes comprised of three alleles were by far the most frequent in the population (49.4%), followed by the genotypes comprised by two (29.6%), four (17.5%), five (2.4%), one (0.5%), and six alleles (0.4%). The association of alleles into genotypes seemed quite random, with a couple of exceptions that grabbed our attention. Here, the pairs composed by the commonest Fuat-DAB*109 allele and either Fuat-DAB*84 or Fuat-DAB*87 (both quite similar in their amino acid sequence) were found in up to 69 different birds (7.6%) and co-occurred more often than expected by random (chi-square test Fuat-DAB*84 and Fuat-DAB*109 = 146.83, 1 df, *P* < 0.0001; Fuat-DAB*87 and Fuat-DAB*109 = 52.95, 1 df, *P* < 0.0001).

**Figure 3:**
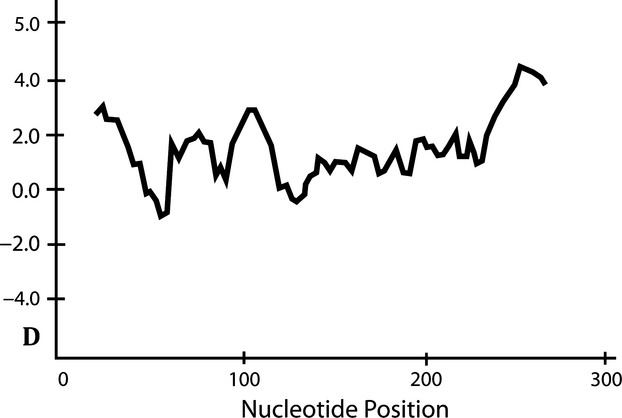
Distribution of Tajima's D value along the coding sequence of exon 2 (Window size = 24 nucleotides; step size = 3 nucleotides).

**Figure 4:**
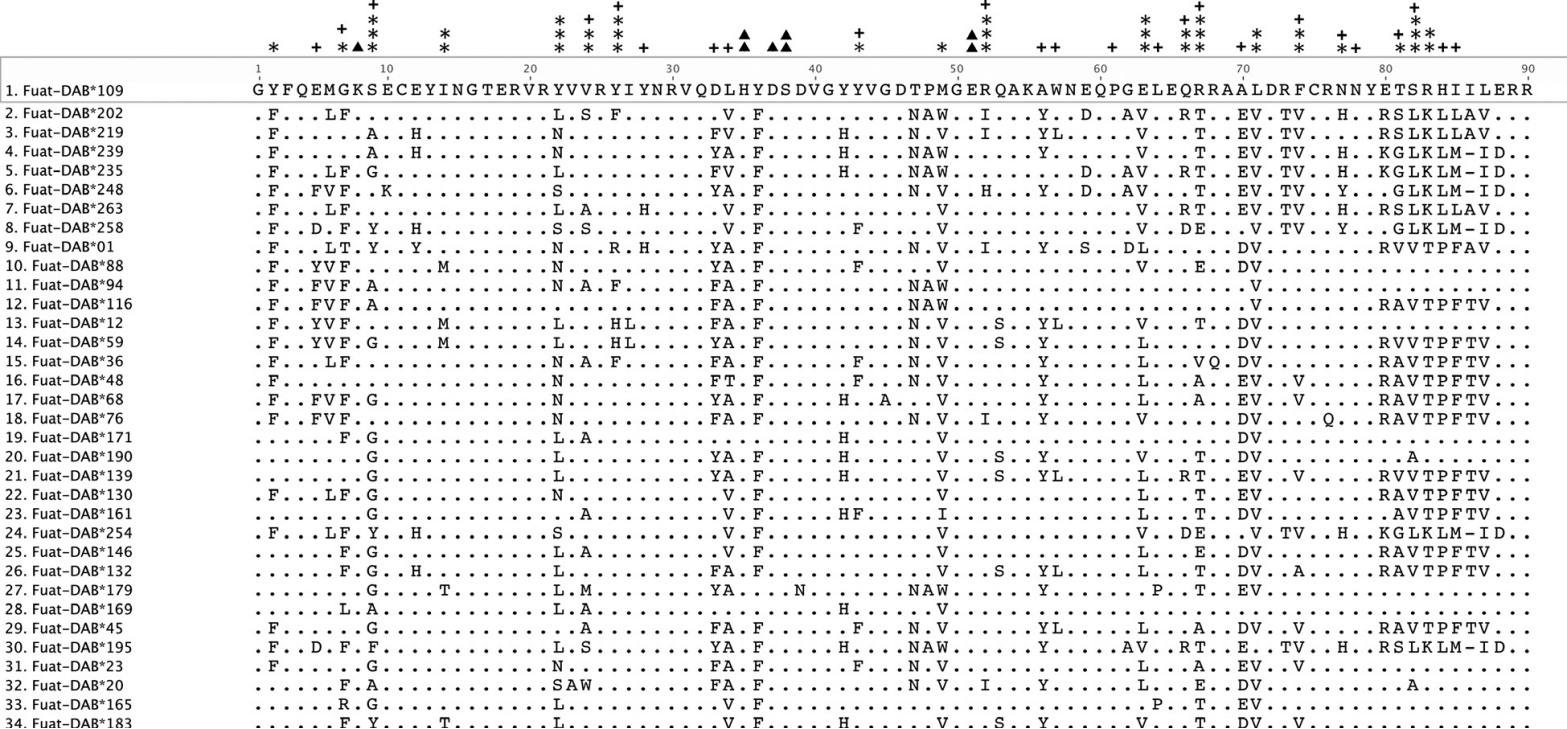
Alignment of the putative amino acid sequences of 34 random exon 2 alleles in the Eurasian Coot. Dots indicate identity with the top sequence (Fuat-DAB*109). Crosses indicate sites known to interact with antigens in the human MHC class II molecule (Brown et al. 1988). Black asterisks and black triangles mark sites under pervasive positive or purifying selection, respectively. The number of asterisks or triangles at a given site is related to posterior probability values (*>0.90, **>0.95, ***>0.99).

The analysis of the 264 putatively functional nucleotide sequences (exon 2) revealed a total number of 107 segregating (variable) sites involving a total of 147 nucleotide substitutions. Average nucleotide diversity was *π* = 0.115, and the average number of nucleotide differences among alleles was *k* = 30.86. We also found a significant excess of high-frequency segregating sites across the alignment of exon 2 sequences (average Tajima's D value = 2.36, *P* < 0.05, see Fig. 3), supporting evidence of balancing selection acting upon these genes. Bayesian inference uncovered pervasive positive, diversifying selection acting upon 19 codons. Five codons showed, on the other hand, pervasive evidence of negative, purifying selection (see Fig. 4). MEGA analysis also corroborated a significant excess of nonsynonymous over synonymous substitution rates specifically for those codons belonging to the PBR (PBR codons: *d*_n_ = 0.361 ± 0.062, *d*_s_ = 0.147 ± 0.062, *Z*-test, *P* < 0.001; non-PBR codons: *d*_n_ = 0.077 ± 0.017, *d*_s_ = 0.063 ± 0.022, *Z*-test, *P* = 0.26). The neighbor-joining tree depicted in Fig. 1A shows that the coot MHC sequences are quite unique with respect to other avian MHC sequences. All coot MHC sequences clustered together, with no evidence of transspecies polymorphisms with respect to other avian groups (Klein et al. 1998). This pattern exhibited by PBR exonic sequences can be also in part attributed to recent gene duplication and/or concerted evolution (e.g., Alcaide et al. 2007; Burri et al. 2008). Finally, the phylogenetic network of exon 2 alleles (Fig. 1B) depicts multiple reticulate events during the evolution of the Eurasian Coot MHC class II B genes (Phi test of recombination; Bruen et al. 2006, mean test value = 0.86, *P* < 0.001), which is in agreement with up to seven recombination breakpoints identified by the SBP method (the partitions lacking decisive recombination evidence are as follows: sites 1–27; 28–78; 79–123; 124–156; 157–180; 181–210; 211–270).

## Discussion

This study describes, to the best of our knowledge, the greatest MHC diversity ever reported in a non-passerine wild bird and also provides the first MHC sequences isolated from an avian species belonging to the Rallidae family. The huge genetic polymorphism reported here is unlikely driven by PCR or sequencing artefacts, as more than 200 alleles were repeatedly found across several birds or independent typing experiments. We are also confident that the 63 alleles found only once in the studied population represent real alleles given that they were found in large number of reads and did not resemble in any case chimeric sequences among co-occurring alleles. In addition, these alleles usually differ in just one or a few amino acid replacements regarding confirmed alleles, and the type of replacement observed is quite widespread within the allele repertoire. Consequently, we have characterized 251 unique amino acid sequences in 902 individuals from a single population of Eurasian Coots, illustrating the large MHC class II variability that may be expected in this species given its nearly cosmopolitan distribution through Europe, Africa, Asia, and Oceania (Cramp 1980) and the marked geographical variation usually exhibited by MHC genes (e.g., Ekblom et al. 2004; Alcaide et al. 2008). MHC genes displaying hundreds of alleles have been previously reported in songbirds (e.g., Bollmer et al. 2012; Sepil et al. 2012) and in a nonpasserine species, the Lesser Kestrel *Falco naumanni* (Alcaide et al. 2008). Therefore, we are confident that our approach is reflecting quite well the actual degree of genetic variability at the Eurasian Coot MHC. Despite the fact that we did not conduct gene expression analyses, evidence supporting both balancing selection and positive selection acting specifically upon antigen-binding sites reinforces the idea that the genes here investigated are functional and expressed (e.g., Bernatchez and Landry 2003; Piertney and Oliver 2006). In addition, studies in non-passerines have shown a large concordance between genomic and transcriptomic approaches with regard to the identification of putatively functional MHC loci, and the reporting of pseudogenes has been rare (e.g., Burri et al. 2008; Cloutier et al. 2011; Strandh et al. 2011; Gangoso et al. 2012).

One surprising finding of the present study was the large proportion of genotypes comprised by three alleles. Many species of non-passerines, such as raptors (e.g., Burri et al. 2008; Agudo et al. 2011) or seabirds (Strandh et al. 2011; Juola and Dearborn 2012), typically display two MHC class II B gene duplicates and hence a maximum of four alleles per individual. In the Eurasian Coot, one would expect a large proportion of tetra-allelic genotypes across a two-gene system, as there are large possibilities to inherit different alleles from each parent given the high variability documented here. The pattern observed in our study can be nonetheless explained by variations in gene copy number among different MHC haplotypes. In fact, this phenomenon has been already documented for the MHC of certain bird species (e.g., Eimes et al. 2011; Strandh et al. 2012) and other vertebrates (e.g., Miller et al. 2010; Siddle et al. 2010). According to the proportions observed, we suggest that the haplotypes comprised of one single MHC gene and those comprised of two genes are the most abundant, being the haplotypes comprised of three genes less common. High rates of gene conversion, through which alleles are frequently shuffled among different gene duplicates, can also add to this phenomenon (e.g., Alcaide et al. 2007; Hosomichi et al. 2008; Promerová et al. 2013). The lack of locus-specific nucleotide motifs within the intron regions flanking exon 2 and the impossibility to assign alleles to locus certainly suggests that gene conversion is operating within the Eurasian coot MHC class II B gene family. However, the extensive allele repertoire makes unlikely that the same allele is shared among different gene duplicates within the same individual (tri-allelic genotypes are also often comprised of three low-frequency alleles), and homozygosity is also expected to be low. Concerted evolution has indeed hindered the investigation of patterns of molecular evolution at individual loci (see some exceptions in Worley et al. 2008; Strand et al. 2013).

Even though our preliminary findings point toward probably one of the most widespread and convincing cases of gene copy variation, allele segregation patterns from parents to offspring and hybridization experiments (i.e., southern blots) must be conducted to raise conclusive evidence regarding this issue. Allele segregation patterns may also shed light about the possibility of postcopulatory selection acting upon allele number in the offspring, for instance through meiotic drive (see for instance Jeffreys and Neumann 2002; Alcaide et al. 2012). Alternatively, elucidating whether the number of MHC alleles can influence fledgling survival would be also of great interest. The Eurasian coot is an abundant species that lays a relatively large clutch size mostly averaging six eggs (e.g., Samraoui and Samraoui 2007) to support research on these topics. We definitively discard that coverage issues are associated with our data on gene copy number variation. Here, similar proportions were obtained when exclusively analyzing a subset of birds from which we obtained at least 500 reads (*N* = 108 birds, average = 892.1 ± 412.3 SD reads per bird; three alleles = 49.1%, two alleles = 26.9%, four alleles = 18.5%, five alleles = 4.6%). The extraordinary diversity uncovered here and the fact that the exon 2 – intron boundaries are usually well conserved due to their participation on the critical process of intron splicing (e.g., Alcaide et al. 2007, 2013) make unlikely that the observed pattern was caused by a high impact of null alleles. Although we are confident that our primers are picking up most of if not all of the variation at the genes here reported, the possibility that other members of the multigene family are not being amplified cannot be ruled out. That said, our estimate of gene copy number per individual (1–3) is largely in agreement with estimates on other non-passerine birds (e.g., Alcaide et al. 2007; Burri et al. 2008; Strandh et al. 2011). Finally, the suspiciously high incidence of some allele combinations (e.g., Fuat-DAB*109 plus either Fuat-DAB*84 or Fuat-DAB*87) would be in agreement with the co-evolution of gene duplicates initially formulated by Kaufman (1999) for the minimal essential chicken MHC. This hypothesis, which suggests the promotion of the most fit allele combinations within the same MHC haplotype so they can be co-inherited, has already found support in wild avian populations such as insular demes of the Egyptian vulture *Neophron percnopterus* (Agudo et al. 2011).

## Conclusion

Extensive genetic polymorphism and gene copy variation, a skewed allele frequency distribution, strong evidence for the action of positive selection and a relative simple and robust genotyping make the Eurasian coot MHC class II and ideal system to investigate the evolutionary implications of MHC variation in wild populations.

## Supporting Information

Data S1 Consensus sequence of intronic sequences flanking the exon 2 of MHC class II B genes as derived from the alignment of Sanger sequencing chromatograms in several individuals.

Data S2 Table showing the number of different birds from which a certain exon 2 allele (amino acid sequence) was retrieved.
