# Supplementary material for: Extraordinary MHC class II B diversity in a non-passerine, wild bird: the Eurasian Coot Fulica atra (Aves: Rallidae)
Source: Ecol Evol. 2014 Feb 13;4(6):688–98. doi: 10.1002/ece3.974 (PMC3967895; doi:10.1002/ece3.974)
Supplement: Data S1 — Consensus sequence of intronic sequences flanking the exon 2 of MHC class II B genes as derived from the alignment of Sanger sequencing chromatograms in several individuals. [file ece30004-0688-sd1.docx]

Consensus sequence for the 3’ distal region of intron 1 in the Eurasian coot MHC class II B genes. Intron 1 sequences are displayed in green and exon 2 sequences in red.

5’- TGCCTGCTGTGTGAGCCCCTGGGGAGGGGGAGCTGCCNCCYCCTGACCRGCCTCCCTGCAYSAGCAGGGTWTTT-3’

Consensus sequence for the 5’ distal region of intron 2 in the Eurasian coot MHC class II B genes. Intron 2 sequences are displayed in blue and exon 2 sequences in red.

5’-GAGAGGAGAGGTGGGTGTRTGGCACAACCCTTCCCTGGGGACAGGCACAAGCYCCRGGCTCCCGGGAGGGAAGAGCACGTCCAGTGCCACACTGTGCCAGGGCCCCRGGCAGCCCCTCACCCTCACCCTGTGTGGGGACAGCRCTGGGCCAGGCGTCCCCTGTTCTCCCCAGTCCTTKCYCAYCCCTCTCTCCCTCTTCCTCCCCACYAGTTCA-3’
